# Mapping *Schistosoma haematobium* for Novel Interventions against Female Genital Schistosomiasis and Associated HIV Risk in KwaZulu-Natal, South Africa

**DOI:** 10.4269/ajtmh.20-0679

**Published:** 2021-05-03

**Authors:** Mahala Livingston, Pavitra Pillay, Siphosenkosi Gift Zulu, Leiv Sandvik, Jane Dene Kvalsvig, Silindile Gagai, Hashini Nilushika Galappaththi-Arachchige, Elisabeth Kleppa, Patricia Ndhlovu, Birgitte Vennervald, Svein Gunnar Gundersen, Myra Taylor, Eyrun F. Kjetland

**Affiliations:** 1 Department of Infectious Diseases Ullevaal, Norwegian Centre for Imported and Tropical Diseases, Oslo University Hospital, Oslo, Norway;; 2 Department of Tropical Medicine, Tulane University School of Public Health and Tropical Medicine, New Orleans, Louisiana;; 3 Department of Biomedical and Clinical Technology, Durban University of Technology, Durban, South Africa;; 4 Discipline of Public Health Medicine, Nelson R Mandela School of Medicine, College of Health Sciences, University of KwaZulu-Natal, Durban, South Africa;; 5 Centre for Clinical Research, Ullevaal University Hospital and Medical Faculty, Oslo, Norway;; 6 BRIGHT Academy, Ugu, South Africa;; 7 Section for Parasitology and Aquatic Pathobiology, Faculty of Health and Medical Sciences, University of Copenhagen, Copenhagen, Denmark;; 8 Institute for Global Development and Planning, University of Agder, Kristiansand, Norway

## Abstract

Women with female genital schistosomiasis (FGS) have been found to have genital symptoms and a three-fold higher risk of HIV infection. Despite WHO recommendations, regular antischistosomal mass drug administration (MDA) has not yet been implemented in South Africa possibly because of the lack of updated epidemiological data. To provide data for future prevention efforts against FGS and HIV, this study explored *Schistosoma haematobium* prevalence in girls and young women and the effects of antischistosomal MDA, respectively. Urinary schistosomiasis and genital symptoms were investigated in 70 randomly selected secondary schools in three districts within KwaZulu-Natal and 18 primary schools. All study participants were treated for schistosomiasis, and schools with the highest urinary prevalence were followed up after 1 and 4 years of MDA. At baseline, urine analysis data showed that most schools were within the moderate-risk prevalence category where biennial antischistosomal MDA is recommended, as per WHO guidelines. Young women had high prevalence of genital symptoms (36%) after correcting for sexually transmitted infections. These symptoms may be caused by infection with schistosomes. However, FGS cannot be diagnosed by urine analysis alone. In KwaZulu-Natal rural schools, this study suggests that antischistosomal MDA with praziquantel could prevent genital symptoms in more than 200,000 young women. Furthermore, it is feasible that more than 5,000 HIV infections could be prevented in adolescent girls and young women by treatment and prevention of FGS.

## INTRODUCTION

Schistosomiasis (Bilharzia) is a waterborne parasitic infection found around the world but predominantly in sub-Saharan Africa, where more than 800 million people are estimated to be currently at risk of infection.^1^ Schistosomiasis is estimated to account for more than 200,000 deaths in this continent annually. This infection is associated with pain and sometimes causes debilitating morbidity in both adults and children.^1^

The most common species of schistosome in Africa is *Schistosoma haematobium*, characterized by urogenital disease.^1^ *Schistosoma haematobium* is notably associated with morbidity of the male and female genitals, bladder, and kidneys.^2^ Adult worms live mainly in the venous plexus surrounding the bladder and genital tissue, depositing eggs in the urogenital tract.^3^ The ensuing host inflammation is composed of immunological cells, fibrosis, and angiogenesis.^3–5^ The female genital manifestations of *S. haematobium*, noted as female genital schistosomiasis (FGS), have previously been reported to affect a mean of 54% (range 33–75%) of women and girls living in schistosome-endemic areas.^6,7^ The presence of schistosome eggs in genital tissue is classically associated with a burning sensation in the genitals, malodorous discharge, and pain as well as infertility, ectopic pregnancies, and miscarriage.^7^

Furthermore, FGS has been associated with a more than 3-fold higher risk of HIV infection in women.^8–11^ Sub-Saharan Africa accounts for approximately 69% of global HIV infections, and the Joint United Nations Program on HIV/AIDS (UNAIDS) reports an HIV incidence of 1.5–2.8% in young women in KwaZulu-Natal.^12^ The two diseases place an already vulnerable population further at risk.^6,9,13^ To improve the sexual and reproductive health outcomes of women and girls, the WHO and UNAIDS together recommend interventions in and research on populations affected by HIV and FGS.^13,14^

Praziquantel is the recommended treatment for schistosomiasis.^14,15^ However, it is effective only in killing adult worms, thereby decreasing egg excretion and deposition, preventing further tissue damage.^16^ The WHO, therefore, recommends routine, early, and repeated treatment for those at risk.^17^ The antischistosomal mass drug administration (MDA) strategies focus almost exclusively on primary school learners aged 6–14 years, although data and treatment among adolescents and young adults are lacking. While many African countries have implemented at least one round of MDA, South Africa, home to the worst HIV epidemic in the world, has not yet implemented universal treatment.^6,15^ There is a lack of epidemiological data on adolescents and young adults in this region, and the consequences of FGS have not been taken into consideration in strategic documents in regard to adolescent health and HIV.^15,18–20^ To provide epidemiological data for use in prevention efforts against both FGS and HIV, this study explored the implications of the natural progression of urogenital schistosomiasis in untreated girls aged 10−12 years and young women aged 16−22 years in KwaZulu-Natal Province, South Africa.

## METHODS

### Study area and design.

**Figure 1.**
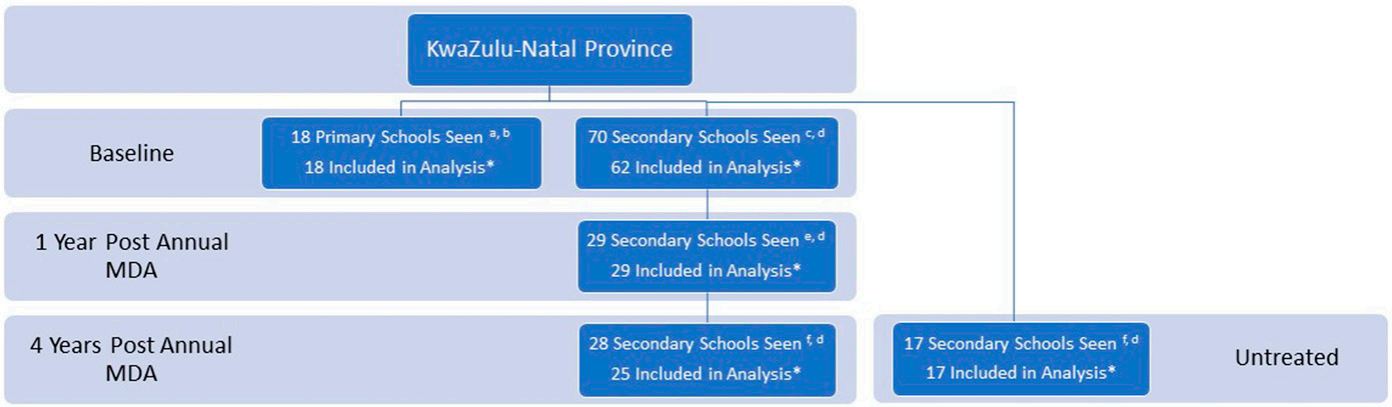
Study population. Legend: ^a^2009. ^b^Ugu district. ^c^Started 2011. ^d^Ugu, iLembe, and Southern uThungulu (King Cetshwayo) districts. ^e^Started 2012, excluding previous schools with low baseline urinary prevalence and low treatment coverage. ^f^Started 2015, excluding previous schools that refused (*n* = 2), with low baseline urinary prevalence. *We excluded primary and secondary schools with fewer than 10 study participants or for which urine results were contaminated or otherwise not available. This figure appears in color at www.ajtmh.org.

According to South Africa’s Department of Education, there are 1,747,010 learners in 4,353 rural schools in KwaZulu-Natal Province.^21^ A cross-sectional study was performed in 70 randomly selected rural secondary schools within three districts along the eastern coastline of KwaZulu-Natal (Figure 1). In young women, urinary and interview data collection began in 2011. Furthermore, approximately 1 year after MDA, follow-up data were collected from the same individuals in 29 of these secondary schools. In 2015, after approximately 4 years of annual MDA, data collection was again performed in 28 of these secondary schools, this time among new learners now populating the schools. To study the effects of antischistosomal treatment, schools with low baseline urinary prevalence and low treatment coverage were excluded from the follow-up investigations.

In addition, in 2015, we included 17 new randomly selected rural, secondary schools within the same districts. These young women had never previously received MDA. Moreover, girls from 18 rural primary schools in Ugu district were also included, as has been described previously.^22^ The study region is known to be endemic for HIV and *S. haematobium*, whereas only occasional cases of *Schistosoma mansoni* have been reported.^23–26^

### Study population.

The research was nested in a larger study on FGS in KwaZulu-Natal.^27,28^ Consenting female participants in secondary schools between ages 16 and 22 years, hereafter referred to as “young women,” were invited to participate in the study. The study excluded those with chronic or severe illness, those who were currently pregnant, and who had no history of sexual activity. Assenting female participants in primary schools between the ages of 10 and 12 years, hereafter referred to as “girls,” were also invited to participate in the study with parental consent.^29^

### Mass drug administration.

Mass drug administration was offered by our team in collaboration with the districts’ departments of health to both male and female individuals in all included schools.^18^ The WHO recommends routine and repeated MDA defined as mass treatment with praziquantel (40 mg/kg) for endemic area schools as, shown in Table 1.

**Table 1:** WHO risk levels and MDA

	High risk	Moderate risk	Low risk
Urinary *Schistosoma haematobium* prevalence	≥ 50%	10–49%	< 10%
WHO recommendations	Annual MDA	Biennial MDA	Two rounds of MDA throughout primary school years

MDA = mass drug administration.

### Laboratory analyses.

#### Urine analysis.

Trained research assistants and school nurses collected urine between 10:00 and 14:00 for three consecutive days from girls included in the study.^22^ For young women, one urine sample was collected.^27^ Samples were transported to the laboratory in dark and temperature-controlled containers. Two 10 mL urine samples were preserved with 1 mL of 2% tincture of Merthiolate in 5% formalin solution. Samples were centrifuged for 10 minutes at 4,000 rpm and microscopically investigated at ×10 magnification for *S. haematobium* eggs. The egg counts were recorded for each 10 mL of urine separately. When more than 1,000 eggs were seen, counting was stopped. Microscopy was performed blinded to previous results and by separate technicians. Quality control was performed by an independent expert on 10% of randomly selected samples.

#### Herpes simplex virus type 2 (HSV-2) and human papillomavirus analyses (HPV).

In young women, samples of cervicovaginal lavage (CVL) were collected during colposcopy by spraying 10 mL of saline on the cervical surface and withdrawing it back into the syringe, as described previously.^30^ Herpes simplex virus type 2 antibodies were detected in serum using ELISA Ridascreen HSV-2 IgG (Davies Diagnostics, Randburg, South Africa) and in ELISA HerpeSelect^®^, IgG assay (Focus Diagnostics, Russelheim, Germany). Herpes simplex virus type 2 PCR was only performed in a subsample of participants because of financial and practical constraints. Likewise, HPV PCR was carried out in a subsample and tested for GP5+/6 + HPV PCR followed by an enzyme immunoassay method using a cocktail mix HPV probes 16, 18, 26, 31, 33, 35, 39, 45, 51, 52, 53, 56, 58, 59, 66, 68, 73, and 82 (Whitehead Scientific, Cape Town, South Africa).^31^

#### *Chlamydia trachomatis* *(CT)* *and Neisseria gonorrhea* *(GC)* analysis.

Cervicovaginal lavage was analyzed using ProbeTec CT/GC strand displacement PCR assay for *Chlamydia trachomatis* and *Neisseria gonorrhea* (Becton Dickinson, Microbiology systems, Johannesburg, South Africa).^28,32^

#### *Trichomonas vaginalis* and HSV PCR.

In-house PCR was used at the Laboratory of Infection, Prevention and Control at the University of KwaZulu-Natal, Durban, South Africa.^33^

#### Syphilis analysis.

Syphilis was detected in thawed serum samples using a Macro-Vue^TM^ test 110/112 (BD, Becton Dickinson Microbiology systems) for a rapid plasma reagin test (RPR) and Immutrep for *Treponema pallidum* hemagglutination assay (Omega diagnostics group PLC, Alva, Scotland, United Kingdom).

#### Bacterial vaginosis analysis.

Nugent’s scoring criteria was used for the diagnosis of bacterial vaginosis*.*^34^ Candidiasis was diagnosed using the Gram staining technique under light microscopy and severity scored (one to five) in accordance with the number of spores seen.^35^ Sexually transmitted infection (STI) analyses were not performed for girls aged 10–12 years.

#### Interviews.

Each school administration provided the names of the associated primary and secondary school(s) in the area. In each school, research assistants invited consenting female study participants to private interviews conducted in the local language, isiZulu.^22,27^ The study participants answered questions regarding their history of genital symptoms and high-risk water contact, defined as regular exposure to potentially infective water bodies covering at least 10% of the body surface, or being in the risk water at least 60 minutes per exposure.^22^ Study participants were asked questions about each year of their lives, including where they lived and whether they might have had contact with freshwater. They were also asked about their housing, laundry, and bathing routines; tap water access; and water outages.

The symptoms, genital burning, abnormal vaginal bleeding, and malodorous discharge, have been reported in girls previously.^22^ By comparison, the same symptoms in the young women were also analyzed. We excluded those who had tested positive for one or more STI, which could otherwise explain their specific symptom or symptoms.^28,30^ Cases with gonorrhea and/or chlamydia were excluded for those who reported bloody vaginal discharge. We excluded cases who were positive for HSV, gonorrhea, chlamydia, *T. vaginalis*, bacterial vaginosis, and/or yeast infection for those who reported malodorous discharge. For those who reported genital burning, we excluded herpes simplex virus, gonorrhea, chlamydia, *T. vaginalis*, and/or yeast infection.

#### Ethics and permissions.

Ethical clearance was provided by the Biomedical Research Ethics Committee (BREC), University of KwaZulu-Natal, 2009 (Ref BF029/07), and from the Department of Health, Pietermaritzburg, KZN, 2009 (Ref HRKM010-08). Regional Ethics Committee Eastern Norway gave ethical clearance on September 17, 2007 (Ref 469–07066a1.2007.535). The Departments of Health and Education in KwaZulu-Natal gave permission. Male and female learners of all participating schools were offered MDA at least once. Care was taken to provide confidentiality for all study participants. Girls provided verbal assent as well as written consent from a parent or guardian prior to participation. Young women provided their written and verbal consent, and parents or guardians of the young women were informed about their participation.

## RESULTS

**Figure 2.**
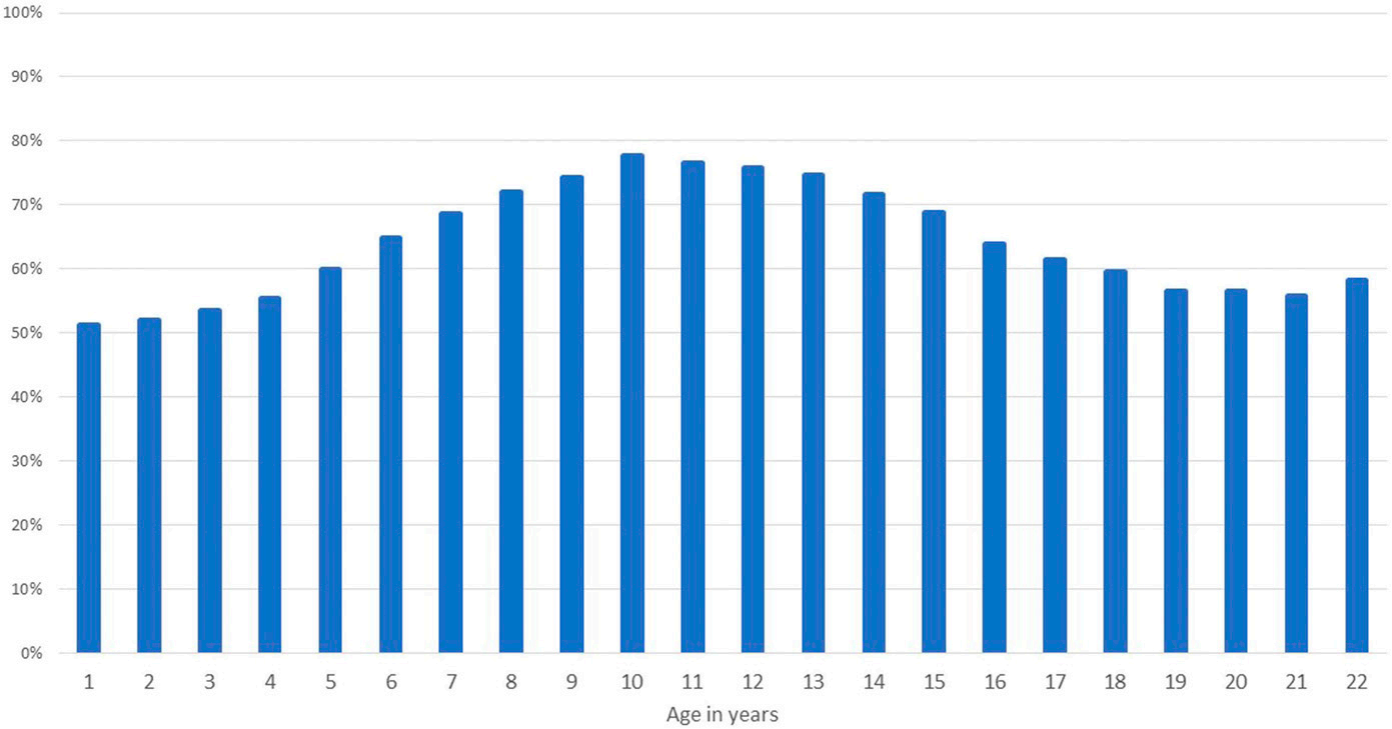
Age vs. risk water contact. Histogram representing percentage of surveyed learners with affirmative risk water contact at given age. The inference on water contact each year was based on where the young woman was staying (anecdotally) and her own or the research staff’s knowledge on when tap water became available in this area, if at all. This figure appears in color at www.ajtmh.org.

**Figure 3.**
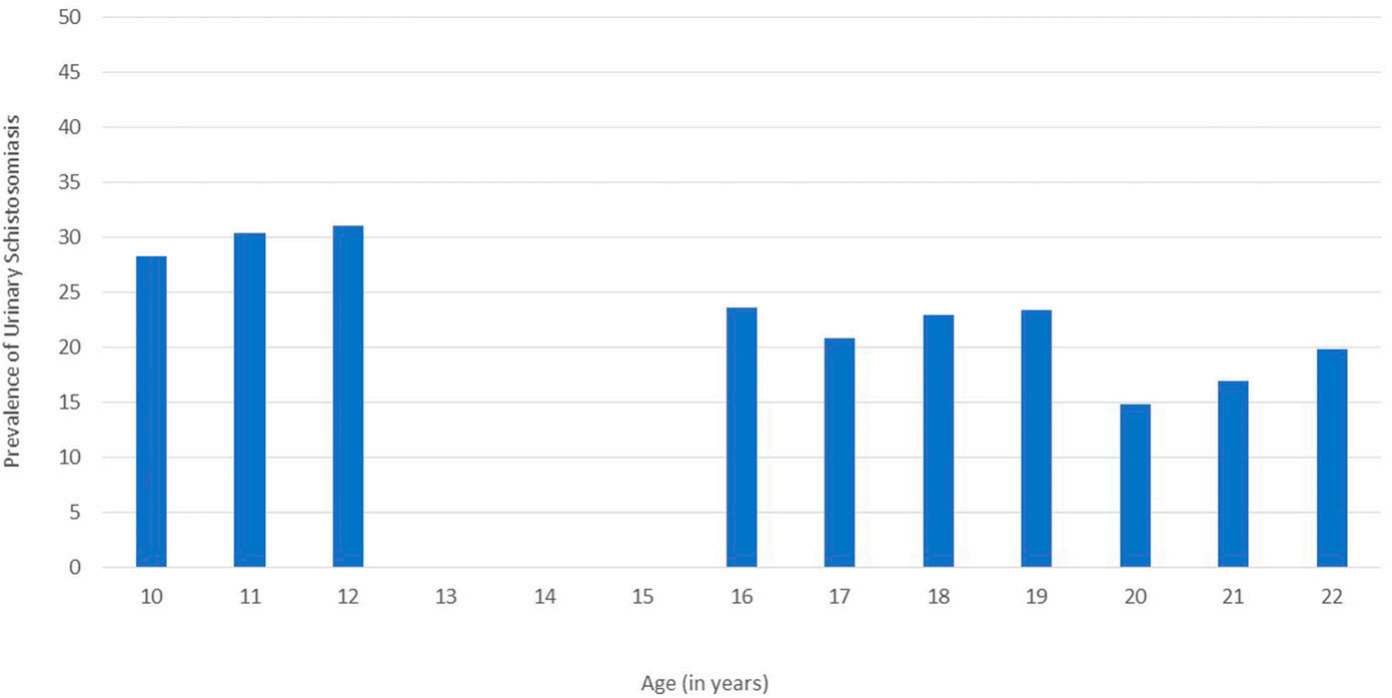
Female urinary schistosomiasis prevalence by age. This figure appears in color at www.ajtmh.org.

At baseline, 3,250 female learners from 18 primary schools and 70 secondary schools participated in the study (Figure 1). The median age was 18 (range 16–22) years in the secondary schools and 11 (range 10–12) years in the primary schools. Figure 2 shows the young women’s likely risk water contact. Contact with potentially contaminated water was highest between ages 8–14 years, peaking at the age of 10 years. River water was the most common type of risk water contact reported among the young women in this study; 93% (2,229/2,387) remembered risk water contact in their lifetime. Tap water was accessible to 96.9% (range 86–100%) of the primary school study participants. As displayed in Figure 3, *S. haematobium* prevalence was highest in the girls, peaking at age 12 years, where over 30% of girls tested urine positive for *S. haematobium*.

### Baseline prevalence rates.

**Figure 4.**
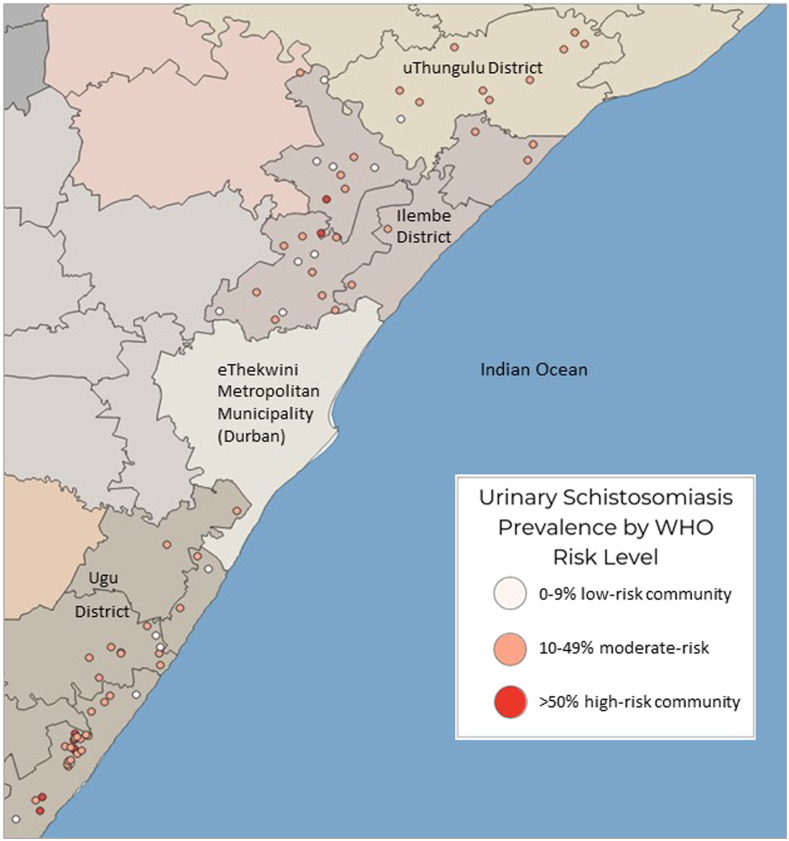
Baseline urinary schistosomiasis prevalence among KwaZulu-Natal primary schools (girls aged 10–12 years) in 2009 and secondary schools (girls aged 16–22 years) in 2011 combined.

As shown in Table 2, approximately three-quarters of the secondary schools had urinary schistosomiasis prevalence between 10 and 49%, qualifying them for biennial MDA per WHO guidelines. Median secondary school prevalence was 20% (range 0–75%), whereas median school prevalence among primary schools was 34% (range 0–61%). As shown in Figure 4, most of the primary schools also fell between 10% and 49% prevalence, and five schools fell above 50% prevalence, qualifying them for annual MDA (Figure 4).

**Table 2:** Risk level findings by urinary *Schistosoma haematobium* prevalence

	High risk (≥ 50%)	Moderate risk (10–49%)	Low risk (< 10%)
Primary schools*	5/16 (31.3%)	9/16 (56.3%)	2/16 (12.5%)
Secondary schools*	2/62 (3.2%)	47/62 (75.8%)	13/62 (20.9%)

* Excluding 2 primary schools and 8 secondary schools that either had fewer than 10 study participants or for which urine results were not available.

### Rural KwaZulu-Natal’s need for MDA.

Extrapolating a conservative estimate of 20.5% urinary schistosomiasis from this study to rural KwaZulu-Natal school children using school data from the South African Department of Education Master Lists,^21^ approximately 329,247 learners are likely to have detectable urinary schistosomiasis (of 1,606,083 rural learners). Furthermore, as per WHO guidelines, approximately 2,625 rural primary schools in KwaZulu-Natal (approximately 934,462 learners) should be offered annual or biennial antischistosomal treatment.

### Baseline genital symptom prevalence.

**Figure 5.**
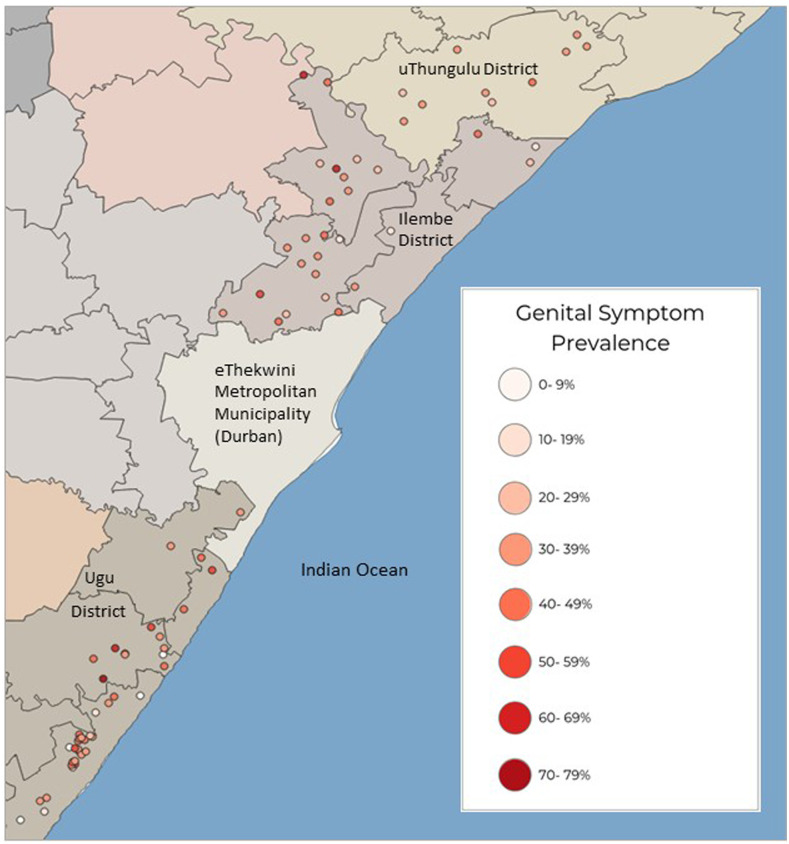
Baseline genital symptom prevalence among KwaZulu-Natal school girls aged 10–12 years and young women aged 16–22 years combined. Legend: The girls and young women were interviewed in 2009 and 2011, respectively.

The median school prevalence for genital symptoms in sexually active 16–22-year-old young women was 67% (range 35–92%) (Figure 5). Further excluding study participants who had one or more sexually transmitted disease(s) that could otherwise cause these symptoms, the median school prevalence for self-reported symptoms in the genitals was 36% (range 13–74%). Nine of the 62 secondary schools had 50% or higher genital symptom prevalence. In girls aged 10–12 years, the median school prevalence for genital symptoms was 30% (range 0–47%).

Extrapolating these prevalence rates to the whole of rural KwaZulu-Natal, approximately 257,056 (32%, range 34,978–450,077) of female learners in this province have genital symptoms, not caused by STIs and hence possibly caused by schistosomiasis.

**Figure 6.**
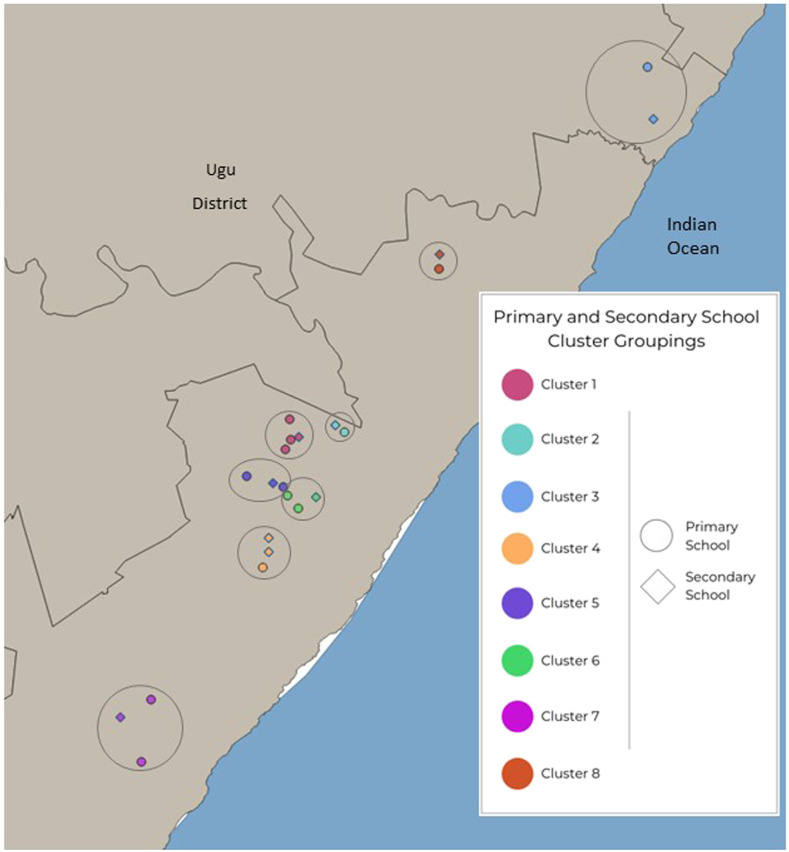
Ugu district primary schools and associated secondary schools displayed in 8 separate color-coded clusters.

**Figure 7.**
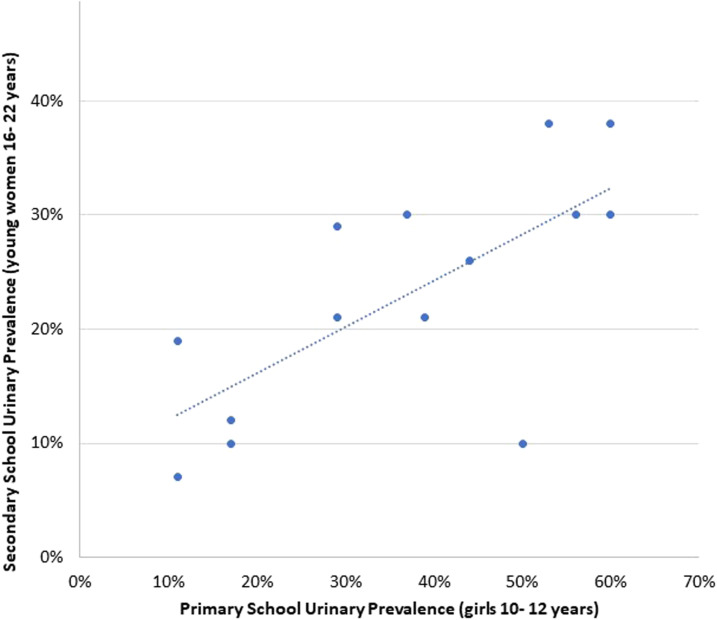
Comparison of urinary schistosomiasis prevalence levels between KwaZulu-Natal 10- to 12-year- old girls and 16- to 22-year-old young women in adjacent primary and secondary schools. This figure appears in color at www.ajtmh.org.

In a subsample in Ugu district, 11 untreated primary schools were paired with the closest associated seven untreated secondary school(s) (Figure 6). Figure 7 shows that prevalence of urinary egg excretion was higher in 10- to 12-year-old girls than young women in neighboring schools (*P* = 0.005). The best fit line for geographically connected areas provides a formula for estimating urinary schistosomiasis prevalence: Secondary school prevalence = Primary school prevalence/2.48 + 7.8%.

In this subsample of 18 schools, there was a trend but no statistically significant association between non-STI secondary schools and in primary school genital symptoms of the same geographical area (*P* = 0.183). Specifically, there was no significant association in reported sensation of genital burning (*P* = 0.232) or malodorous discharge (*P* = 0.065).

### Analysis of rivers contacted.

**Figure 8.**
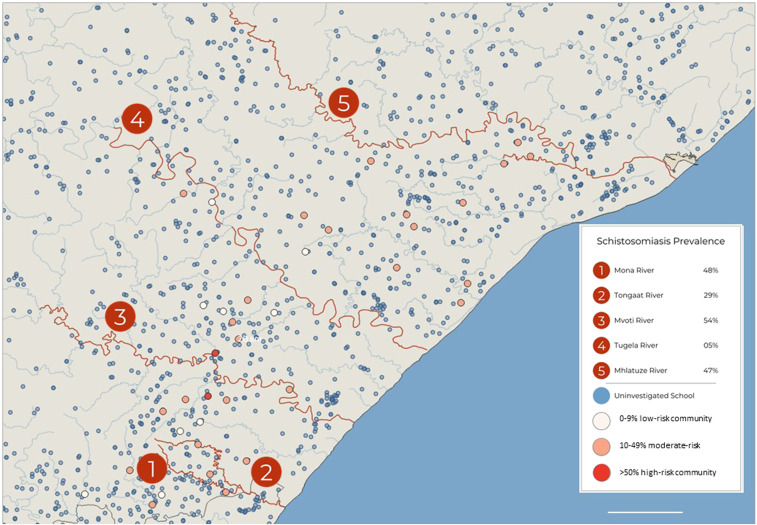
Rivers mentioned by young women aged 16–22 years and their secondary schools and uninvestigated schools. Legend: Mona, Tongaat, Mvoti, Tugela, and Mhlathuze rivers mentioned by 46, 31, 33, 43, and 63 individuals, respectively; prevalence calculated among those who mentioned contact with each river.

Approximately 42% (993/2350) of young women were able to provide the name(s) of the rivers or other water bodies with which they were most often in contact, if any. As shown in Figure 8, more than 50% of those who mentioned the Mvoti River had detectable urinary schistosomiasis qualifying them for annual MDA. There are approximately 30 schools along Mvoti in iLembe district; only two were included in this study, and both qualified for MDA per WHO recommendations, annually and biennially, respectively. Figure 8 shows that schools along the same river may fall in different MDA management categories. Likewise, it can be seen from the map that even schools relatively close to each other, for example, within a 5-km radius, may land in different WHO treatment categories. Notably, all the tested schools in iLembe were secondary schools; most of the other schools are primary schools. As shown in Figure 7, these primary schools would likely have higher prevalence of schistosomes than indicated on the map.

### One and 4 years after annual MDA.

**Figure 9.**
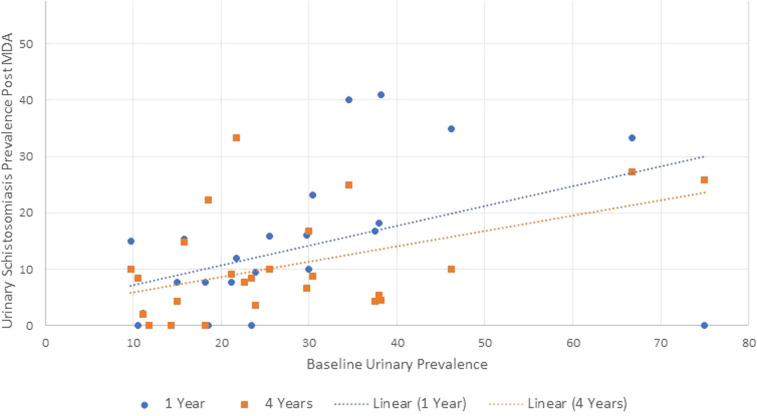
Urinary schistosomiasis after treatment in young women in KwaZulu-Natal. Legend: Dots and blue hatched line: Individuals seen after 1 year (2012) were the same as those seen at baseline (2011). Diamonds and orange hatched line (2015): same schools but different pupils from baseline. This follow-up excluded 4 schools which had fewer than 10 study participants.

The young women aged 16–22 years were investigated again approximately 1 year following the first MDA. On this follow-up, nearly half of the schools (13/29 or 44.8%) had fallen below the 10% prevalence level after just one MDA effort (Figure 9).

These schools were investigated again following 4 years of annual MDA, with a new group of young women now populating each school. After four rounds of mass treatment, 18/25 (72%) of the schools were now below the 10% urinary prevalence level and none of the schools had remained above 50% prevalence.

### Potential effects of MDA on the HIV epidemic.

If 329,247 of rural learners in KwaZulu-Natal have detectable urogenital schistosomiasis, we may presume half of these are female, 164,624. If 54% (range 33–75%) of these have FGS, it would follow that 88,897 female learners in KwaZulu-Natal Province will exhibit current or developing FGS symptoms and signs. Table 3 shows that more than 5,300 HIV seroconversions could be prevented annually by adequate treatment and prevention of *S. haematobium* in girls and young women.

**Table 3:** Annual preventable HIV incidence and FGS prevalence in KwaZulu-Natal

	Calculation	Count
Estimated FGS prevalence^6,7^	54% (range 33–75%)	88,897
Conservative HIV incidence estimate^12^	2%	1,778
Conservative HIV incidence in FGS positive^9,11,36^	6%	5,334

FGS = female genital schistosomiasis.

## DISCUSSION

In accordance with the WHO recommendations, we have found that almost one million children are in need of antischistosomal treatment in KwaZulu-Natal and more than 2,600 schools in this province qualify for annual or biennial MDA. If such interventions were implemented, this study indicates that 5,300 annual incidents of HIV transmission could be prevented among these adolescent girls and young women.^6,9–12^ Despite access to tap water in much of the region, more than 90% of the participants in this study reported to have had risk water contact and more than 30% reported genital symptoms that could not be otherwise explained by STIs.

As in previous studies, our findings suggest that the morbidity of *S. haematobium* infection does not necessarily decrease with age but may become more difficult to detect through urine sampling.^7,37^ Other studies have shown that schistosome egg excretion in the urine is highest between the ages of 10 and 15 years.^37^ However, circulating anodic antigen (CAA) levels indicate that adults also have live worms. After the age of 15 years, urinary egg excretion begins to decline, despite CAA levels indicating a relatively high worm burden.^37–39^ This warrants MDA among secondary learners in endemic areas. Moreover, such MDA efforts would be best strategized based on the detected prevalence rates in the primary schools, as primary school prevalence appears to show a more accurate representation of geographical burden of *S. haematobium*.

Several studies have indicated that treating schistosome infections in endemic areas of sub-Saharan Africa could be a novel approach to reducing HIV incidence.^11,36,40–42^ Female genital schistosomiasis has been found to be associated with higher frequencies of monocytes and the HIV receptor CCR5, both of which decrease following antischistosomal treatment with praziquantel.^28^ Furthermore, schistosomiasis/HIV coinfection has been found to increase HIV-1 viral loads in HIV-1–infected individuals, potentially accelerating AIDS development by up to 3 years.^11^ With approximately six million individuals infected with schistosomiasis in South Africa, routine and repeated MDA could have an important effect on transmission, morbidity, and mortality from both infections.

The South African Department of Health and the WHO recommend 75% treatment coverage in schistosome-endemic area schools.^17,43^ However, only a few ad hoc mass drug interventions have been implemented, and seldom have these campaigns met adequate treatment coverage.^18^ In 2011, treatment coverage was just less than 45% (range 14.5–82.6%).^18^ Of those who did not receive treatment, the majority were reported to be secondary school learners aged 15–24 years and disproportionately male. Before this small pilot effort, it had been 14 years since the last mass praziquantel treatment campaign in South Africa.^23^

There is currently no method to accurately distinguish between early- and late-stage schistosome infection, and the *S. haematobium* parasites can survive as long as 30 years.^2^ Consequently, it is not possible to know whether infected young women contracted schistosomiasis near their secondary school recently, or their primary school years previously.^16^ In our data analysis, behavioral differences between primary girls and young women suggest higher risk of contact with contaminated water between ages 8 and 14 years, peaking at age 10 years. It is therefore quite likely that many positive young women had become infected with *S. haematobium* as children, several years before. This is an important epidemiological consideration as most South African learners attend primary and secondary schools in different locations, often several kilometers apart.^21^ The Department of Education Lists show approximately three times more primary schools than secondary schools, necessitating secondary learners to commute further distances to their respective schools.^21^ In determining the exact locations of the contaminated water sources, it should also be noted that schistosomiasis transmission is highly focal.^44^ Our data indicate large differences in prevalence among schools within relatively close distance. For this reason, it might be useful to determine treatment frequency by considering the primary school as a point of reference for the decisions regarding interventions.

### Limitations.

We collected one urine sample from young women, and it is likely that school prevalence rates would have been higher had multiple samples been collected from each individual.^27^ Furthermore, we did not look at intensity of *S. haematobium* in this population; this might have provided useful information. This study includes data from two cohorts; only 18 primary schools were included, and for financial reasons, it was not possible to include girls aged 13–15 years. Most children reported to have access to tap water for drinking purposes. However, there a few recreational facilities for swimming in rural KwaZulu-Natal, and children continue to swim in infested waters even if they have taps.^29^ Furthermore, after the recruitment was completed, it is possible that environmental changes, such as increased access to tap water, may have occurred within this region, changing infection incidence patterns. However, if individuals become infected in childhood, it is likely they remain infected, without antischistosomal treatment, regardless of any environmental changes which may have occurred since their initial infection. Extrapolated calculations for the estimated overall urogenital schistosomiasis and genital symptom prevalence among KwaZulu-Natal learners are based on the assumption of uniform age and gender distributions in other rural areas, but this could not be confirmed.^45,46^ Furthermore, these calculations are based on data from the Department of Education, which only include enrolled learners. Children and young adults who are not enrolled in school are perhaps more infected than those who do attend school, through their domestic chores. The true prevalences may therefore be higher for genital symptoms, urinary schistosomiasis, and HIV incidence.

### Conclusion.

By conservative estimates, treating for urogenital schistosomiasis infections in endemic areas of this region could cost-effectively prevent more than 53,000 new cases of HIV the next decade.^6,47^ Our data indicate most of the tested primary and secondary schools within the Ugu, iLembe, and uThungulu districts of KwaZulu-Natal fall in the moderate-risk schistosomiasis category and would require biennial antischistosomal MDA per WHO guidelines.^17^ We would recommend mass treatment of both primary and secondary school learners not only to avoid FGS in adolescents but also because of the impact that this may have on HIV prevention in this population. We further suggest that frequency of MDA in secondary schools should be based on the prevalence category of the associated primary schools.
